# Machine learning models for predicting prostate cancer recurrence and identifying potential molecular biomarkers

**DOI:** 10.3389/fonc.2025.1535091

**Published:** 2025-02-17

**Authors:** Maria Eliza Antunes, Thaise Gonçalves Araújo, Tatiana Martins Till, Eliana Pantaleão, Paulo F. A. Mancera, Marta Helena de Oliveira

**Affiliations:** ^1^ Graduate Program in Biometrics, Instituto de Biociências de Botucatu (IBB), Universidade Estadual Paulista (UNESP), Botucatu, São Paulo, Brazil; ^2^ Department of Biodiversity and Biostatistics, Instituto de Biociências de Botucatu (IBB), Universidade Estadual Paulista (UNESP), Botucatu, São Paulo, Brazil; ^3^ Institute of Biotechnology, Universidade Federal de Uberlândia (UFU), Patos de Minas, Minas Gerais, Brazil; ^4^ Laboratory of Clinical and Experimental Pathophysiology, Instituto Oswaldo Cruz (IOC), Rio de Janeiro, Rio de Janeiro, Brazil; ^5^ School of Computing, Universidade Federal de Uberlândia (UFU), Patos de Minas, Minas Gerais, Brazil; ^6^ Institute of Mathematics and Statistics, Universidade Federal de Uberlândia (UFU), Patos de Minas, Minas Gerais, Brazil

**Keywords:** supervised learning, artificial intelligence, next generation sequencing, molecular markers, prognostic

## Abstract

Prostate cancer (PCa) recurrence affects between 20% and 40% of patients, being a significant challenge for predicting clinical outcomes and increasing survival rates. Although serum PSA levels, Gleason score, and tumor staging are sensitive for detecting recurrence, they present low specificity. This study compared the performance of three supervised machine learning models, Naive Bayes (NB), Support Vector Machine (SVM), and Artificial Neural Network (ANN) for classifying PCa recurrence events using a dataset of 489 patients from The Cancer Genome Atlas (TCGA). Besides comparing the models performance, we searched for analyzing whether the incorporation of specific genes expression in the predictor set would enhance the prediction of PCa recurrence, then suggesting these genes as potential biomarkers of patient prognosis. The models showed accuracy above 60% and sensitivity above 65% in all combinations. ANN models were more consistent in their performance across different predictor sets. Notably, SVM models showed strong results in precision and specificity, particularly considering the inclusion of genes selected by feature selection (*NETO2*, *AR*, *HPN*, and *KLK3*), without compromising sensitivity. However, the relatively high standard deviations observed in some metrics indicate variability across simulations, suggesting a gap for additional studies via different datasets. These findings suggest that genes are potential biomarkers for predicting PCa recurrence in the dataset, representing a promising approach for early prognosis even before the main treatment.

## 1 Introduction

Prostate cancer (PCa) poses a global public health challenge with high mortality. In 2024, an estimated 300,000 new cases of PCa will be diagnosed, representing about 15% of all global cancers and with a projected 35% mortality rate (1). According to 2022 GLOBOCAN data, it is the fourth most diagnosed cancer globally (7.3%) (2). Primary PCa risk factors include age, family history, genetic traits, obesity, diet, and lifestyle conditions (3–5). Additionally, socio-environmental and socioeconomic factors play a role in cancer occurrence, with ethnicity and geographic location as relevant aspects (6). The Prostate-Specific Antigen (PSA) test is a widely used biomarker for diagnosing and monitoring PCa. PSA levels ≥ 4.0 ng/mL are considered elevated, potentially indicating abnormalities. Disease prognosis is based on the Gleason score and tumor staging, which assess the tumor’s histological grade, extent, and spread. Post-treatment, patient follow-up includes PSA measurements every three to six months (7).

Biochemical recurrence affects 20% to 40% of patients and is marked by rising PSA levels (8). However, this rise does not necessarily signal cancer return, as PSA lacks specificity, unable to distinguish between aggressive and non-aggressive tumors, leading to high false-positive rates (9, 10). Given that PSA results vary per patient (11, 12), there is a need to optimize predictions to improve diagnostic accuracy and reduce recurrence rates. In fact, the heterogeneity, plurality, plasticity and complexity of PCa make an assertive therapeutic approach difficult. Surgery and radiotherapy are adopted for localized tumors. However, late diagnosis is frequent and metastatic disease requires systemic therapies that, for the most part, are not curative (13). In this context, improved prognostic and predictive tools are necessary to overcome the challenges related to PCa, such as: differentiation of lethal and non-lethal disease; personalized therapeutic, and accessibility, especially in low-income countries (14).

In recent years, many studies have explored PCa diagnosis using machine learning (ML) algorithms, particularly through imaging techniques (15). Some models have also identified radiomic, genomic, and clinical biomarkers for PCa diagnosis (9, 16). However, fewer studies have focused on disease recurrence prediction using ML, especially with molecular biomarkers. Deng et al. (17) applied five ML models to predict PCa in patients with low PSA, achieving high performance with Random Forest (RF). Liu et al. (18) used ML to predict Gleason score upgrades, with Lasso-regularized Logistic Regression (Lasso-LR) performing best. Similarly, Lee et al. (19) tested multiple models for biochemical recurrence prediction, with Gradient Boosting Machines (GBM) showing the highest performance.

Machine learning (ML) algorithms have been extensively used to predict both the diagnosis and prognosis of various cancers. Zhou et al. (20) assessed the predictive performance of five ML models in forecasting recurrence in gastric cancer patients post-surgery, with the logistic regression model demonstrating the highest accuracy. Furthermore, these algorithms facilitated the identification of critical factors associated with recurrence, including body mass index (BMI), operation time, age, among others (20). For glioblastoma, ML models have been developed with high accuracy in predicting disease recurrence and mortality, utilizing diverse types of data ranging from imaging and genetic profiles to demographic details (21). Kim et al. (22) proposed clinically applicable prognostic prediction models for glioblastoma multiforme, estimating overall survival and progression-free survival. The Random Survival Forest (RSF) model exhibited the best performance, but all models successfully stratified high-risk recurrence groups for glioblastoma multiforme up to 5 years.

Despite these advances, predicting PCa recurrence remains challenging and less defined than diagnosis prediction. Recent research has targeted potential molecular biomarkers, such as mRNAs, microRNAs, lncRNAs, and repetitive sequences, to enhance disease prediction. Wang et al. (23) identified six omic biomarkers that differentiated high- and low-risk recurrence groups. Tong et al. (24) used ML techniques to identify and validate biomarkers associated with PCa prognosis through protein-protein interaction networks. These studies underscore ML’s potential in improving PCa prognosis, though further research is needed to refine recurrence prediction using molecular features.

Given previous study limitations and the need to enhance recurrence predictions, this study aims to compare three supervised ML models—Naive Bayes (NB), Support Vector Machines (SVM), and Artificial Neural Network (ANN)—in predicting PCa recurrence. We also investigate whether adding potential molecular biomarkers to traditional clinical features (PSA levels, Gleason score, and tumor staging) can boost model performance. The data and models used in this study contribute to the development of tools that address key biological features of PCa and can be adapted to investigate other tumors, particularly those with a worse prognosis. The paper is structured as follows: Section 2 describes the methodology, including the dataset, preprocessing, and the models and metrics used for comparison. Section 3 presents the results of feature selection and model performance. Section 4 discusses the findings and potential applications.

## 2 Methodology

**Figure 1:**
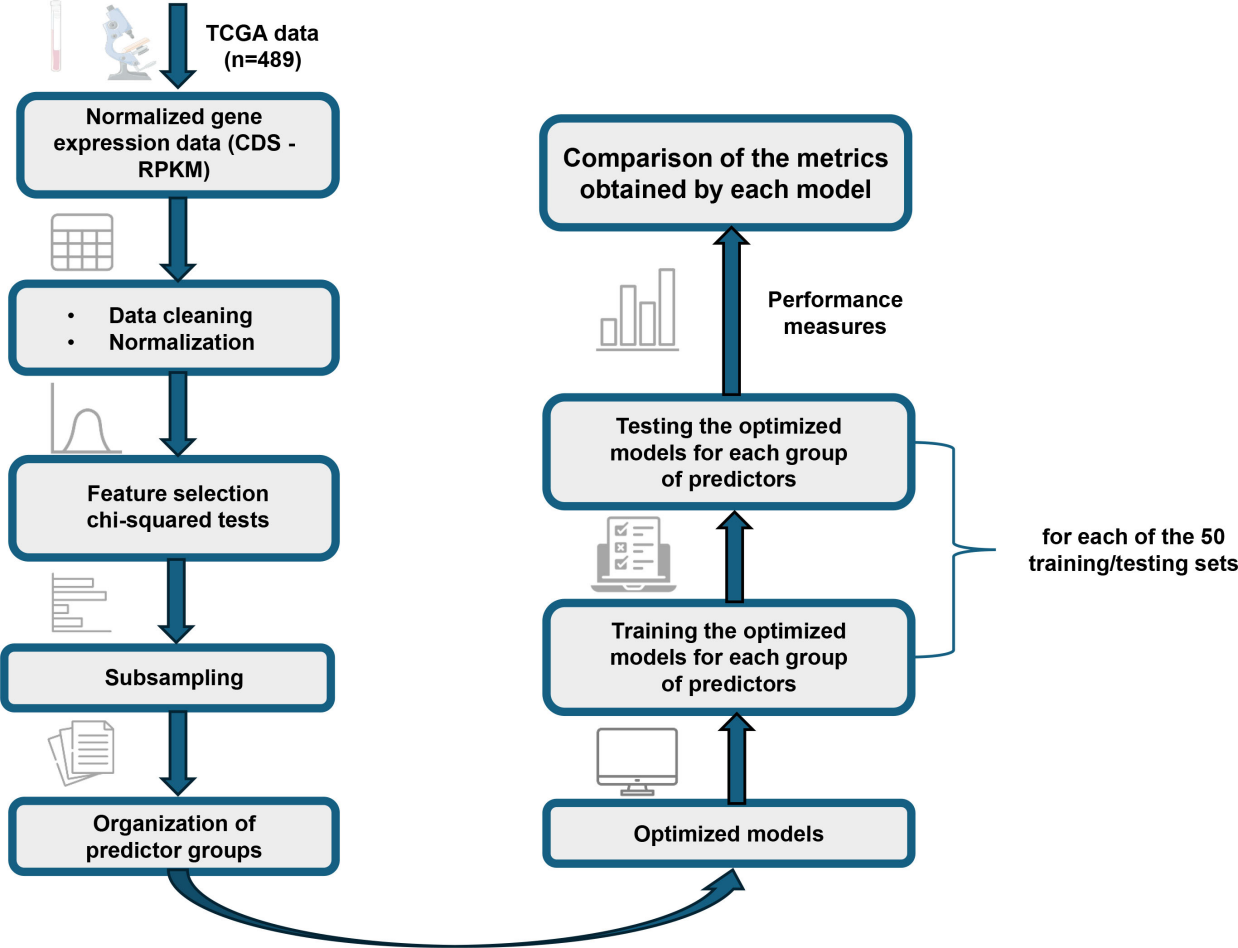
Detailed flowchart of the methodology applied in this study. The diagram illustrates the main steps, from the processing and organization of gene expression data from TCGA to the optimization and comparative evaluation of NB, SVM, and ANN models.

An overview of the methodology and analyses employed in this study is presented in **Figure 1**. The applied methodology is described in the following subsections.

### 2.1 Database

To construct classification models and identify potential new biomarkers for predicting PCa recurrence events a dataset of 489 patients with the disease available from The Cancer Genome Atlas (TCGA)^1^, accessed in January 2023, was used. The expression of the genes *KLK3*, *AR*, GSTM3, *NETO2*, *HPN*, *PRUNE2*, and *FOLH1*, as provided in the RNA-seq version 2 files (Illumina Hi-Seq), was evaluated. Each gene expression profile (tag count per gene - CDS) was normalized to reads per kilobase of exon per million mapped sequence reads (RPKM), according to the CDS length and total tag count using the following relation:


(1) $$\frac{10^{9}\;\times \;C}{\left(N\;\times \;L\right)},$$

where 10^9^ is a correction factor, *C* is the number of reads corresponding to a gene, *N* is the total number of mappable tags in the experiment, and *L* is the CDS length (25).

**Table 1** presents the attributes of the selected database. In addition to gene expression data, information already used in the clinic for PCa diagnosis and prognosis was also considered: preoperative serum PSA level, Gleason score, and tumor staging. The staging is nominal, thus a value between 0 and 1 was assigned to each stage (T1 to T4). For the class of interest “recurrence events”, a value of 0 was assigned to non-recurrence events and 1 to recurrence events. Of the 489 patients, only 419 were used because of missing information in some attributes. Among these, 85 presented cancer recurrence events, whereas 334 did not.

**Table 1:** Attributes and range of values of prostate cancer data.

Attributes	Range of values
Serum PSA levels (ng/mL)	4 - 107
Gleason Score	6 - 10
Tumor Staging	0.4 - 1
*KLK3*	310.81 - 43729.25
*AR*	0.05 - 24.31
*GSTM3*	0.45 - 39.80
*NETO2*	0.08 - 13.08
*HPN*	0.29 - 612.77
*PRUNE2*	0.03 - 62.53
*FOLH1*	0.58 - 914.05
Class	no-recurrence-events, recurrence-events

As shown in **Table 1**, the attributes have different value ranges. Therefore, all data were normalized between 0 and 1 using the following equation:


(2) $$x_{norm}=\frac{x\;-\;x_{min}}{x_{max}\;-\;x_{min}},$$

to improve training characteristics and prevent attributes with high values from exerting a greater influence on the classification (26, 27).

To eliminate the effect of class imbalance, a subsampling process was applied to the dataset, consisting of the random selection of an adequate number of samples from the majority class (0) and equalizing the number of samples among classes. This ensures equitable representation during modeling. After this rearrangement, 90% of the data were used for training and validation, while 10% were reserved as a completely independent test set (28). Within the training data, a 10-fold cross-validation scheme was applied to tune the hyperparameters and evaluate the models performance. This ensures that the hyperparameter optimization process does not involve the independent test set, thereby preventing overfitting to already analyzed data. This random subsampling process was repeated 50 times, creating 50 different training and testing datasets. Each data point was used for validation exactly once across all iterations, ensuring robust and representative results. All model simulations were performed on each of these 50 datasets, ensuring representativeness and robustness of the analysis.

For the simulations, the *Classification Learner* toolbox (29) in MATLAB © ^2^ was used for feature selection and optimization of the models, which will be described in the next section. Due to the limited availability of data, and consequently the training data in this study, we decided to use these three models, which are effective in different contexts. The NB classifier generally exhibits good binary classification accuracy, which aligns with the problem addressed here (30). SVM, on the other hand, is widely used in pattern recognition problems and tends to deliver good results (31), although the appropriate choice of hyperparameters and kernels is crucial for optimal performance, especially with small datasets. Additionally, ANN were included due to their ability to capture complex non-linear relationships in the data, which may not be easily modeled by simpler algorithms (26).

### 2.2 Naive-Bayes classifier

The NB method attempts to solve the problem of predicting a class based on a vector of *d* features using generative hypotheses (32, 33). To realize this, it assumes (naively) that, given a class, features are independent of each other. That is, considering y ∈ {0,1} as the classes and $x=\;\{x_{1},x_{2},\ldots ,x_{d}\}$ as the feature vector, where each $x_{i}\in \;\{0,1\}$ , we obtain


(3) $$P[X=\text{x}|Y=y]=\prod_{i=1}^{d}\;P\left[X_{i}=x_{i}|Y=y\right].$$

To describe the probability function presented in Equation 3, 2*^d^* parameters are required, meaning that the number of examples required for classification increases exponentially with number of features. To identify an optimal classifier, Bayes theorem can be used, which defines the probability of an event *A* occurring given that *B* has occurred. Considering the theorem and Equation 3, the optimal Bayes classifier $\left(h_{Bayes}\left(x\right)\right)$ can be defined as class *y* that makes the expression (Equation 3) the most probable (maximum possible) for feature vector *x*, that is,


(4) $$h_{\text{Bayes}}(x)=\underset{y\in \{0,1\}}{\text{argmax }}P[Y=y]\prod_{i=1}\;P[X_{i}=x_{i}|Y=y].$$

For each class, the classifier estimates the probability of a given feature belonging to that class and selects the value of *y* that maximizes the expression. The generative hypothesis of the classifier reduces the number of parameters learned by the model, which is very advantageous (33). NB is a simple and effective technique then it can be applied to a range of problems, including disease prediction, such as cancer, because it has a good ability to handle complex datasets, which allows for robust analysis (34).

### 2.3 Support vector machine

SVM is a classification algorithm that seeks to find the optimal separating hyperplane for a dataset controlling the complexity of the models by selecting important data (the so-called support vectors) to construct the separation surface (35, 36). SVM models are built around a function (kernel) that transforms input data in an n-dimensional space to obtain the best-separating hyperplane. The models decision function is fully defined by the support vectors, which are the data points closest to the hyperplane (28).

SVM emerged (37) as a solution to circumvent situations where the training error of classification was low; however, the test error was high, indicating poor generalizability to unseen data. The formulation of SVM is typically presented as a quadratic programming problem and when the data are not linearly separable, the algorithm penalizes violations with loss terms or uses kernel tricks to construct nonlinear separation surfaces (35, 36). A limitation of the proposed algorithm is the high computational cost of the training and testing phases (27). Despite this, SVM is used in machine learning models to predict cancer development and prognosis because it is simple to interpret and provides a sparse solution, making it advantageous over other approaches (34).

### 2.4 Artificial neural network

ANN is a graph-based structure composed of interconnected units, known as nodes, which mathematically model the behavior of biological neurons. These nodes are connected by unidirectional or bidirectional edges, where the weights represent the strength of the connections between units. Inspired by the biological model of neurons, the weights of these connections simulate the influence of synapses inhibiting or facilitating the transmission of signals between neurons (33, 36).

The neural network processes input data through specialized input nodes, transmitting it through hidden layers to generate outputs via dedicated output nodes. A node can serve both input and output functions, depending on its role within the network. The design of an ANN is typically divided into two phases: training and testing. During the training phase, the network is trained to predict outputs based on given input data. In the testing phase, the network is evaluated to determine whether to halt or save the training and it is then used to make predictions on unseen data (36).

For classification models, an ANN consists of three main components: (i) the neural model, which describes how each node processes input to produce output; (ii) the network architecture, which defines the connections between nodes; and (iii) the training algorithm, which adjusts the connection weights to optimize the model. The learning process aims to estimate the set of weights that allow the network to perform classification tasks optimally by minimizing a classification error metric. Each class of networks offers a specific learning scheme tailored to its architecture (33, 36).

Proposed in the mid-20th century, neural network learning has become an effective machine learning paradigm. This methodology has been successfully applied to a wide range of real-world classification problems across various domains. In particular, ANNs have shown significant potential in improving the accuracy of disease detection and predicting patient outcomes, thereby contributing to more informed clinical decision-making (26, 28, 33).

### 2.5 Metrics

The performance of each model, for different groups of attributes, was evaluated using the following metrics derived from confusion matrix information: true positives (*T P*), true negatives (*T N*), false positives (*F P*), and false negatives (*F N*). The metrics evaluated in, the context of binary classification problems are, described below.

#### 2.5.1 Sensitivity

Sensitivity is defined as the true positive rate obtained from the classifier (38), that is,


(5) $$\text{Sensitivity}=\frac{TP}{TP\;+\;FN}.$$

#### 2.5.2 Specificity

Specificity is the complementary metric to sensitivity because, it is defined as the rate of true negatives identified by the classifier (38), that is,


(6) $$\text{Specificity}=\frac{TN}{FP\;+\;TN}.$$

#### 2.5.3 Accuracy

Accuracy is defined as the fraction of instances correctly classified by the classifier, whether they are true positives or true negatives (28, 38), that is,


(7) $$\text{Accuracy}=\frac{TP\;+\;TN}{TP\;+\;TN\;+\;FP\;+\;FN}.$$

#### 2.5.4 Precision

Precision is defined as the accuracy at which the classifier can correctly classify positive examples (28, 38), that is,


(8) $$\text{Precision}=\frac{TP}{TP\;+\;FP}.$$

#### 2.5.5 AUC

The receiver operating characteristic (ROC) curve is a graphical tool that provides a summary visualization of the performance of learning algorithms in relation to varying decision criteria, typically in binary classification scenarios. It helps identify regions of optimal behavior, facilitates model selection, and allows for comparison of learning algorithms. In this context, the AUC measures the probability that the classifier will assign a higher score to a randomly chosen positive example than to a randomly chosen negative example. It always ranges between [0, 1], with the upper bound obtained by a perfect classifier. A reasonable classifier performance is indicated by an AUC greater than 0.5 (28, 38).

## 3 Results

In this section we present the results obtained from the feature selection, the performance of the models and their respective predictions in the tests.

### 3.1 Feature selection

The feature selection procedure aims features from the dataset reducing the number of required variables while retaining as much relevant information as possible for the problem classification. Two features may individually carry valuable information classification, however their combining into a feature vector with high mutual correlation may offer little additional benefit. Furthermore, a high number of features are directly related to a high number of classifier parameters, which can be computationally disadvantageous. Therefore, keeping the number of features as small as possible aligns with the optimized predicting classifiers and good generalization capabilities (27).

One possible step in feature selection is to analyze each biomarker independently and test its categorizing ability for the given problem, thereby avoiding the use of elaborated techniques involving unnecessary computational effort. This analysis can be performed using hypothesis tests (27). Thus, the feature selection algorithm used in this study was the univariate feature classification using a chi-square test. The algorithm checks whether each predictor variable is independent of the response variable using individual chi-square tests and then it ranks features based on chi-square tests statistic *p*-values. The scores provided by the proposed algorithm correspond to −log(*p*) (39).

**Figure 2:**
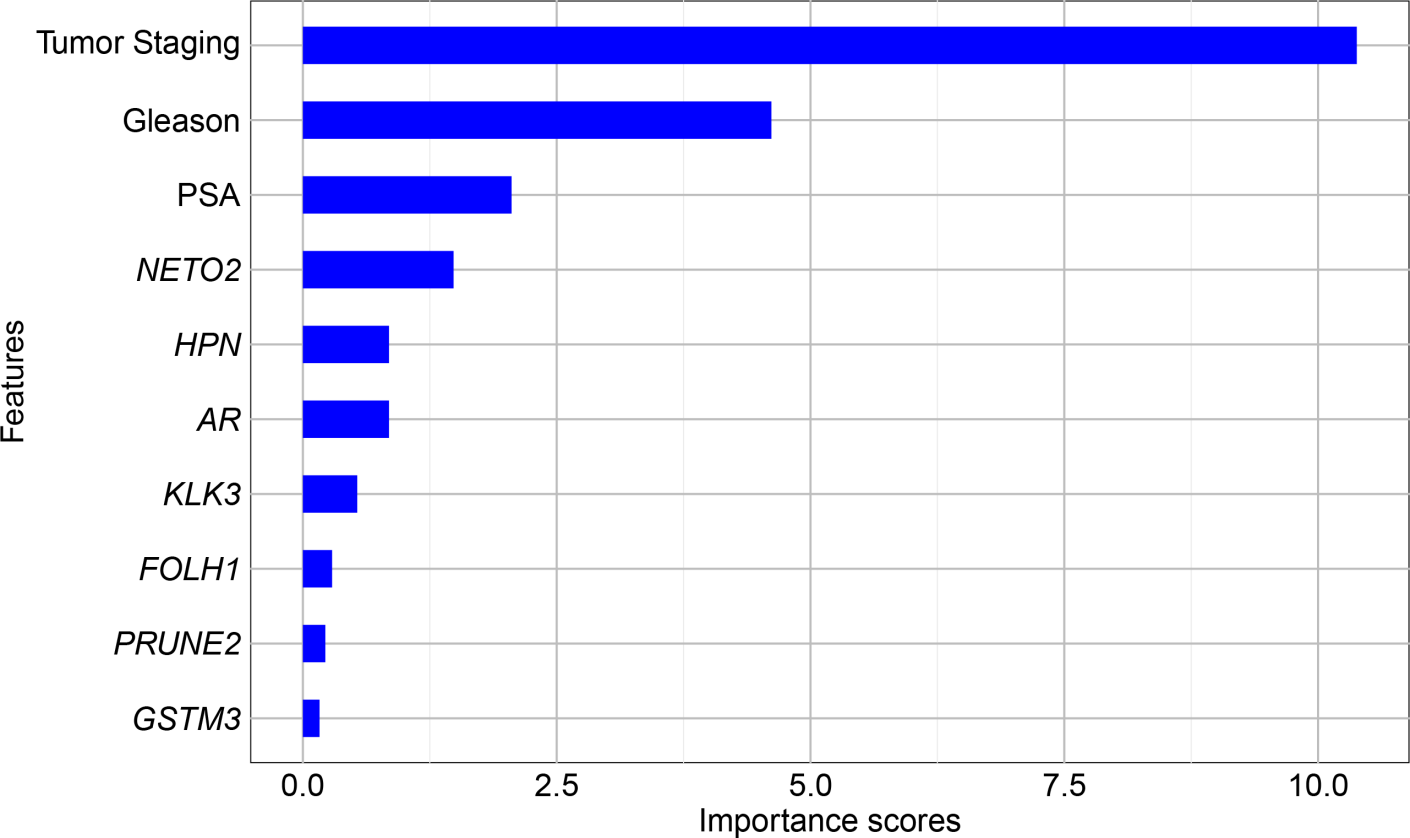
Univariate feature ranking for classification using chi-square tests.

**Figure 2** illustrates the ranking of the attribute contribution for the classification models. As expected, the top three attributes contributing to classification are tumor staging, Gleason score, and preoperative PSA level, which are already used clinically for disease diagnosis and prognosis (40). In addition, following this sequence, the prominent genes were *NETO2*, *HPN*, and *AR* (the latter two with the same importance score), as well as *KLK3*. Other genes had scores below 0.5 and were not considered relevant for the classification of PCa recurrence in this dataset.

Using the results from feature selection, six different predictor groups were organized aiming to identify the best combination for classification. The composition of these groups is presented in **Table 2**.

**Table 2:** Groups of predictors used in the optimized NB, SVM and ANN models.

Group	Predictors
1	Serum PSA, Gleason Score and Tumor Staging
2	Serum PSA, Gleason Score, Tumor Staging, *NETO2* and *AR*
3	Serum PSA, Gleason Score, Tumor Staging, *NETO2* and *HPN*
4	Serum PSA, Gleason Score, Tumor Staging, *NETO2* and *KLK3*
5	Serum PSA, Gleason Score, Tumor Staging, *NETO2*, *AR* and *HPN*
6	Serum PSA, Gleason Score, Tumor Staging, *NETO2*, *AR*, *HPN* and *KLK3*

### 3.2 Optimized models

With the predictors defined, the optimized models were obtained using the *Classification Learner* to enhance the performance of the classification algorithms. This technique attempts to determine the hyperparameters of the models, which are settings that must be defined prior to training and they are not learned from the data (41). The hyperparameter optimization process was carried out exclusively on the training data, using a 10-fold cross-validation scheme to evaluate and select the best combination of hyperparameter values for each model. For a given type of model, the software tests various combinations of hyperparameter values using an optimization scheme that searches to minimize the classification error of the algorithm and returns a model with the optimized hyperparameters (42).

For the NB classifier, the optimized hyperparameters were the distribution types (called Distribution names), and the Kernel types (Gaussian, Box, Epanechnikov, or Triangle). For the SVM, the optimized hyperparameters included the kernel function (Gaussian, linear, quadratic, or cubic), the box constraint level and the kernel scale being both positive values on a log scale between [0.001, 1000]. Finally, for the ANN, networks were used with only one fully connected layer, where the hyperparameters to be optimized were the size of this layer (integers on a log scale in the range [1,300]), the activation function (ReLU, Tanh, None, and Sigmoid), and the regularization strength λ (real values on a log scale in the range [0.00001/n, 100000/n], where n is the number of observations) (42). In all cases, the optimization method was grid search, using 10 as the division of each grid.

To ensure robustness, the final models were evaluated using an independent test set that had not been used during training or hyperparameter optimization. In total, 18 models were obtained - three for each predictor group - comprising 6 NB classifiers, 6 SVM, and 6 ANN. These results represent the average performance metrics obtained across 50 simulations, where each simulation used a distinct pair of training and testing datasets created through the random subsampling process described earlier. **Table 3** presents a detailed comparison of NB, SVM, and ANN results for each of the proposed models.

**Table 3:** Mean (± standard deviation) of the metrics (in %) across 50 different simulations of the models for each predictor group, with the highest mean values highlighted in bold.

	Models	Accuracy	Sensitivity	Specificity	Precision	AUC
1	NB	66.00 (± 9.55)	87.00 (± 11)	45.00 (± 16.17)	61.86 (± 8.14)	68.05 (± 13.25)
	SVM	67.75 (± 9.89)	**89.50** (± 12.21)	46.00 (± 15.24)	62.80 (± 7.56)	**74.27** (± 13.84)
	ANN	68.25 (± 12.43)	78.75 (± 16.61)	57.75 (± 19.38)	66.15 (± 12.30)	73.55 (± 13.78)
2	NB	67.75 (± 12)	69.75 (± 16.38)	65.75 (± 18.70)	68.38 (± 13.56)	71.53 (± 14.10)
	SVM	67.50 (± 10.79)	84.50 (± 13.96)	50.50 (± 19.72)	64.25 (± 10.26)	71.63 (± 14.19)
	ANN	68.25 (± 13.47)	74.25 (± 15.24)	62.25 (± 19.64)	67.57 (± 13.57)	73.13 (± 14)
3	NB	65.63 (± 9.39)	85.75 (± 12.37)	45.50 (± 16.51)	61.78 (± 7.96)	66.11 (± 11.73)
	SVM	69.88 (± 12.80)	71.25 (± 16.41)	**68.50** (± 16.41)	**70.06** (± 12.86)	73.22 (± 13.30)
	ANN	68.13 (± 12.89)	75.25 (± 16.27)	61.00 (± 18.84)	66.88 (± 12.43)	73.25 (± 13.89)
4	NB	66.25 (± 10.34)	86.50 (± 12.84)	46.00 (± 16.64)	62.14 (± 8.75)	66.58 (± 12.25)
	SVM	69.63 (± 12.69)	75.25 (± 17.77)	64.00 (± 17.97)	68.60 (± 12.54)	73.19 (± 14.55)
	ANN	68.25 (± 13.17)	75.00 (± 15.97)	61.50 (± 18.36)	67.10 (± 12.94)	72.75 (± 14.27)
5	NB	65.25 (± 9.71)	85.75 (± 13.60)	44.75 (± 17.33)	61.52 (± 8.45)	64.58 (± 12.57)
	SVM	68.38 (± 11.74)	82.50 (± 16.94)	54.25 (± 19.17)	65.33 (± 11.12)	73.13 (± 13.89)
	ANN	67.38 (± 12.89)	77.75 (± 15.42)	57.00 (± 19.91)	65.58 (± 12.77)	72.22 (± 13.90)
6	NB	64.88 (± 9.18)	85.00 (± 13.60)	44.75 (± 16.19)	61.23 (± 7.87)	65.05 (± 12. 13)
	SVM	**70.00** (± 12.69)	76.25 (± 17.17)	63.75 (± 18.08)	68.76 (± 12.52)	72.94 (± 14.18)
	ANN	66.00 (± 13.19)	75.25 (± 16.65)	56.75 (± 18.59)	64.28 (± 12.56)	71.19 (± 14.41)

## 4 Discussion

### 4.1 Performance of the NB models

All NB models demonstrated accuracy, sensitivity, precision, and AUC values above 60%. However, the specificity of these models exceeded 60% only for predictor group 2, which includes *NETO2* and *AR* genes. This model also achieved the highest accuracy, precision, and AUC, representing the best performance among the NB classifiers for this data set.

When evaluating classifier performance, balancing multiple metrics is crucial, particularly in binary classification problems. Among the NB classifiers, 5 of the 6 predictor groups presented a sensitivity of over 80%, but specificity remained under 50%, which is suboptimal from a clinical perspective. Accuracy alone is not sufficient as the primary evaluation metric, since classification errors carry different significance levels (28). For instance, false negative cases in predicting PCa recurrence may lead to undiagnosed and untreated disease, whereas false positive diagnoses could result in unnecessary treatments and interventions, both of which have significant clinical implications (43).

### 4.2 Performance of the SVM models

The SVM models demonstrated accuracy and precision higher than 60% for all predictor groups. Sensitivity and AUC were also higher than 70% for all combinations. Specificity was lower than 50% only for group 1, which did not include any genes as predictors. In contrast, for the other combinations, specificity exceeded 50%, surpassing 60% in predictor groups 3, 4, and 6, all of which featured different combinations of genes. For example, comparing the specificity of group 3 (68.50%) with group 1 (46%) reveals an increase of 22.5%. Moreover, group 3 demonstrated the highest specificity among all predictors, maintaining a sensitivity above 70%. Groups 4 and 6 also exhibited a strong balance among metrics, with group 6 achieving the highest accuracy of all predictors. These results indicate that incorporating a combination of genes as predictors can significantly enhance the prediction of disease recurrence in this model.

### 4.3 Performance of the ANN models

When comparing the results obtained from ANN, a notable similarity can be observed among the respective metrics for each group, which was less evident in the previous models. All models exhibited accuracy above 65%, with sensitivity and AUC exceeding 70%. The specificity and precision metrics were also over 60%, in contrast to the specificity observed in NB and SVM models. Group 1 demonstrated the highest sensitivity and AUC, achieving the same accuracy as groups 2 and 4, which included genes as predictors. Furthermore, group 2 exhibited the highest specificity and precision among the ANN models, with an increase of 4.5% and 1.42%, respectively, compared to the group without genes.

### 4.4 Impact of molecular biomarkers on model performance

In general, the ANN metrics were more consistent among the various groups of predictors. In Group 3, which includes the *NETO2* and *HPN* genes, both SVM and ANN classifiers achieved metrics exceeding 65%. The SVM model demonstrated strong results in precision and specificity among all combinations tested, without compromising sensitivity, reinforcing its robustness for clinical applications. However, the relatively high standard deviations observed, particularly for specificity and precision in Group 3 (SVM: 68.50 ± 16.41 and 70.06 ± 16.82, respectively), indicate variability across simulations. This variability suggests that results could differ with another dataset. In Group 4, SVM and ANN models again demonstrated strong performance in sensitivity, specificity and precision. Nonetheless, the variability, as reflected in the standard deviations (e.g., SVM specificity: 61.13 ± 12.27), underscores the need for further optimization to ensure consistent model behavior. These observations highlight the importance of addressing variability by leveraging larger datasets to ensure more robust and reliable model performance.

The inclusion of genes selected by the feature selection process significantly enhanced the models performance, particularly in terms of specificity and precision. Preoperative serum PSA levels, tumor staging, and Gleason score, and also including the *NETO2* gene, remained consistent for all six predictor groups, underscoring their primal role in classification. From a clinical perspective, assessing the expression of these genes could facilitate earlier predictions of disease recurrence in advance of primary treatment, offering considerable benefits with the management and treatment planning for the patients. While the standard deviations suggest a gap for improvement, these results highlight the potential of integrating gene expression data to refine predictions in future studies.

Recent studies have highlighted the role of the *NETO2* gene in many types of cancer, including prostate cancer (PCa) (44, 45), demonstrating its potential in predicting recurrence. The gene *HPN* has been associated with tumor invasion and metastasis (46, 47), while the androgen receptor (*AR*) gene is crucial in promoting metastatic cancer progression (48, 49). Additionally, the *KLK3* gene has been extensively studied for its role in PCa prognosis and recurrence prediction (50, 51). While a standardized protocol to predict cancer recurrence remains elusive, machine learning (ML) tools, particularly supervised learning models, offer a valuable way for advancing research in this area. These models are accessible, easy to implement, and enable the exploration of variable importance, integrating diverse clinical data to improve both diagnosis and prognosis.

As studies continue to explore the significance of these biomarkers, integrating gene expression with clinical data via machine learning could facilitate earlier, more accurate predictions of disease recurrence. The results from this study emphasize the importance of incorporating *NETO2*, *HPN*, *AR*, and *KLK3* as part of a comprehensive model for prostate cancer prognosis, pointing towards their potential to improve clinical outcomes.

## Data Availability

The original contributions presented in the study are included in the article/supplementary material. Further inquiries can be directed to the corresponding author.

## Footnotes

1
https://portal.gdc.cancer.gov

2 License 41081750.
